# Supplementary material for: Mid-infrared single-photon 3D imaging
Source: Light Sci Appl. 2023 Jun 9;12:144. doi: 10.1038/s41377-023-01179-2 (PMC10256700; doi:10.1038/s41377-023-01179-2)
Supplement: Supplementary file 1 — Supplementary Information [file 41377_2023_1179_MOESM1_ESM.pdf]

Mid-infrared single-photon 3D imaging
- Supplementary Information -

Jianan Fang,[1] Kun Huang,[1,2,3,*] E Wu,[1,2] Ming Yan,[1,2] and Heping Zeng[1,2,4,5,6,†]

[1]*State Key Laboratory of Precision Spectroscopy, East China Normal University, Shanghai 200062, China*

[2]*Chongqing Key Laboratory of Precision Optics, Chongqing Institute of East China Normal University, Chongqing 401121, China*

[3]*Collaborative Innovation Center of Extreme Optics, Shanxi University, Taiyuan, Shanxi 030006, China*

[4]*Chongqing Institute for Brain and Intelligence, Guangyang Bay Laboratory, Chongqing, 400064, China*

[5]*Shanghai Research Center for Quantum Sciences, Shanghai 201315, China*

[6]*Jinan Institute of Quantum Technology, Jinan, Shandong 250101, China*

Supplementary Note 1: Details about experimental setup

The detailed schematic for the experimental setup is illustrated in Supplementary Figure 1. The whole system is comprised of two main parts: synchronous dual-color pulse preparation and mid-infrared (MIR) upconversion 3D imaging. The involved light sources originate from an ytterbium-doped fiber laser (YDFL) and an extended cavity diode laser (ECDL), which spectrally centered at 1030 and 1550 nm, respectively. The YDFL is a mode-locked fiber laser in a polarization-maintaining architecture, which can deliver ultrafast pulses at the repetition rate about 21.6 MHz. The average power is augmented to 5 W by using two-stage fiber amplifiers. The pulse duration of amplified pulses is compressed to 270 fs, as measured by an optical auto-correlator (APE, pulseCheck). The output power is divided into two branches by a polarization beam splitter (PBS). The splitting ratio can be controlled by varying the orientation angle of the half-wave plate (HWP). One portion is spatially combined via a dichroic mirror (DM1) with the amplified light from the continuous-wave ECDL. The mixed beams are then focused into a periodically poled lithium niobate (PPLN) crystal with a length of 25 mm. The difference-frequency generation (DFG) is performed to prepare mid-infrared light source at 3070 nm. The generated MIR pulse is self-synchronized with the pump pulse at the 1030 nm, which provides a simple and robust alternation to optical parametric oscillators [1, 2]. The prepared synchronized pulses at two disparate wavelengths are essential in the subsequent implementation of coincidence-pumping upconversion imaging [3–5]. The PPLN crystal is placed in an oven, and the temperature is stabilized at 48 °C to approach the phase-matching condition for a poling period of 30.3 μm. The MIR power can reach to 50 mW at presence of average powers of 1 W and 100 mW at 1030 nm and 1550 nm, respectively. The high-power MIR beam facilitates the alignment and calibration of the imaging system. Note that the MIR illumination is intensively attenuated in the case of demonstrating imaging performances at

*Electronic address: khuang@lps.ecnu.edu.cn
†Electronic address: hpzeng@phy.ecnu.edu.cn

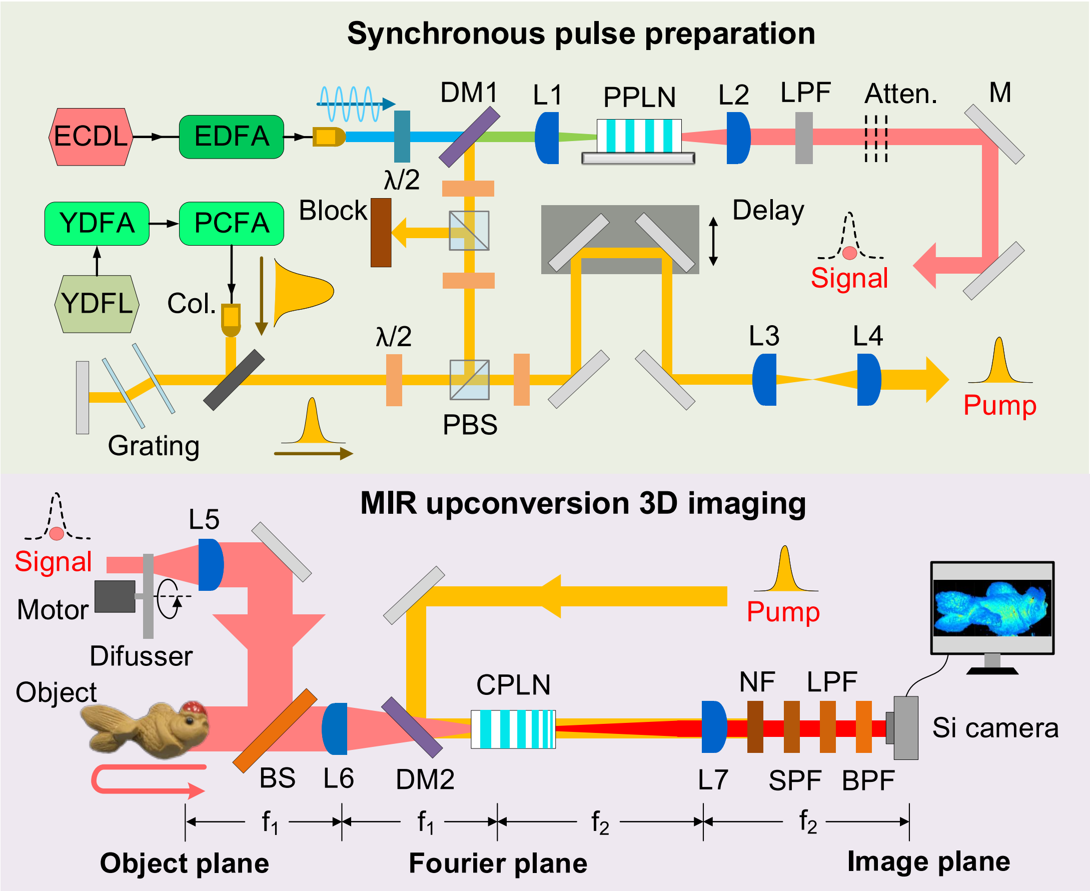

Supplementary Figure 1: Schematic for the experimental setup. Top panel presents the fiber laser system to prepare synchronized dual-color ultrafast pulses, which serve as the signal and pump sources. Bottom panel shows the time-resolved and wide-field imaging system based on coincidence-pumping upconversion detection. ECDL: extended cavity diode laser; YDFL: ytterbium-doped fiber laser; PPLN: periodically poled lithium niobate crystal; CPLN: chirped-poling niobate crystal; EDFA: Er-doped fiber amplifier; YDFA: Yb-doped fiber amplifier; PCFA: photonic crystal fiber amplifier; Col: collimator; DM: dichroic mirror; L: lens; Atten: neutral density attenuator; M: silver mirror; $\lambda/2$: half-wave plate; BS: beam splitter; PBS: polarizing beam splitter; NF: notch filter; LPF, SPF and BPF: long-, short-, and band-pass filter.

the low-light level. The pulse duration of the MIR pulse is inferred to be 295 fs from the measured cross-correlation trace between the MIR and pump pulses.

Subsequently, the prepared MIR signal along with the other portion of the amplified YDFL output is steered into the upconversion 3D imaging section. A spinning ground-glass diffuser (Thorlabs, ED1-C20-MD) is inserted in the MIR beam path in order to eliminate the speckle effect arising from the spatial coherence of the laser source [1]. The diffuser is mounted on a motor with a maximum rotation speed of 200 cycles per second. The illumination power distributed onto a centimeter-size object is estimated to be about 1% of the laser power before the diffuser, which takes into consideration of the insertion loss of optical elements and the deflection angle of the beam. Since the depth imaging is arranged in a reflective configuration, the diffused MIR light

is reflected onto the targeted scene by a beam splitter. The back-scattered infrared photons are collected into a 4f imaging system consisting of two relay lenses with focal lengths of 50 mm and 100 mm, respectively. A chirped-poling lithium niobate (CPLN) crystal is placed at the Fourier plane, where the spatial frequency components are spectrally upconverted through the sum-frequency generation (SFG). The size of CPLN crystal is 3×2×10 mm^3. The poling period linearly ramps from 16 to 24 μm along the axial direction. The use of the CPLN crystal permits to increase the acceptance angle [4], thus leading to a larger field of view than that for PPLN crystals [3, 5]. The upconverted image at 771 nm is captured by an electron multiplying charge-coupled device (EMCCD, Andor, iXon Ultra 888). The conversion efficiency is estimated to 0.4% at the pump power of 2.3 W. The limited efficiency, as the penalty of achieving wide-field and high-resolution imaging, is ascribed to a loosely focused pump beam size of 2 mm and a short interaction length within the CPLN crystal. To suppress the pump-induced fluorescence noises, the upconverted light passes through a series of spectral filters, including a notch filer (Thorlabs, NF1030-45), a short-pass filter (Edmund Optics, #64335), a long-pass filter (Edmund Optics, #68653), and a band-pass filter (Edmund Optics, #88014). The total transmission is calculated to be about 80%, and the rejection ratio at the pump wavelength is estimated to be 210 dB. The high-contrast noise filtering is essential to realize the single-photon imaging performance. The time-resolved operation required for the three-dimensional (3D) imaging is realized by temporally scanning the translational stage (Thorlabs, NRT150/M) in the pump path. The optical pulsed pump provides an ultrashort timing gate for the reflected MIR photons. Consequently, the time-stamped images are used to retrieve the structure and reflectivity information of the scene [4, 5].

The dark noise is an important figure of merit for sensitive optical imaging. In contrast to conventional MIR imagers based on HgCdTe and InSb, the silicon-based cameras are featured with an extremely low dark current due to the much larger bandgap. The dark current of the EMCCD is specified to be about 8.7×10^{-4} electrons/pixel/second. In our experiment, the background noise of the upconversion imaging system is mainly contributed by the pump-induced fluorescence. To characterize the imaging sensitivity, a noise equivalent power at the entrance of the MIR upconversion imager can be defined as

$$N_{\rm noise} = \frac{N_{\rm p}\times M}{\eta_{\rm QE}\times G}\times\frac{1}{\eta_{\rm conv}\times\eta_{\rm filter}}\ , \tag{1}$$

where $N_{\rm p} = 500$ counts/pixel/second is the pump-induced noise count, $\eta_{\rm QE}$=80% is the quantum efficiency at 771 nm for the EMCCD camera, M=4.93 electrons/count is the A/D conversion factor, G=1000 is the gain factor, $\eta_{\rm conv}$=0.4% is the conversion efficiency, and $\eta_{\rm filter}$=80% is the total filtering efficiency. The given parameters result in $N_{\rm noise}=10^3$ photons/pixel/second.

Supplementary Note 2: Temporal walk-off effect on conversion efficiency

The coincidence pumping technique is used to implement the nonlinear frequency upconversion in our experiment. The involved ultrashort pump not only favors to improve the conversion efficiency due to the high peak power, but also helps to reduce the background noise within a narrow time window [2, 6]. Furthermore, the femtosecond pump pulse serves as an ultrafast optical gate

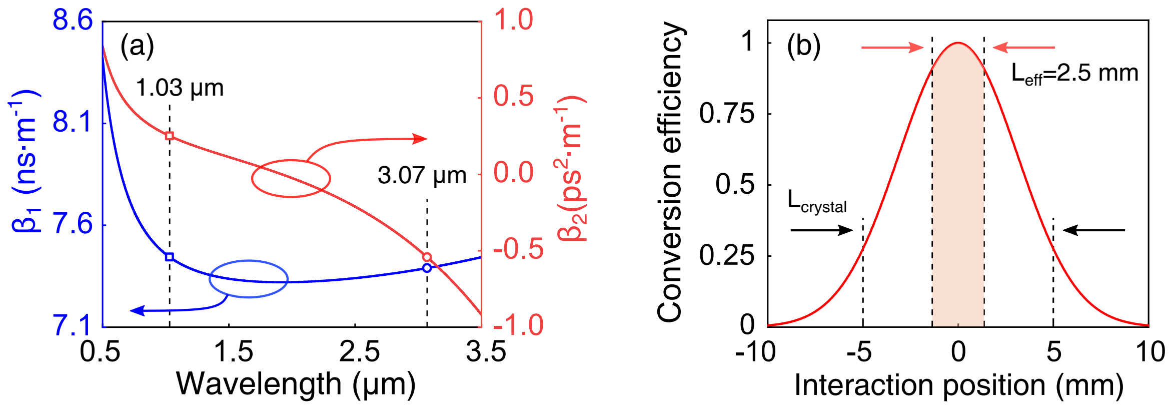

Supplementary Figure 2: Numerical simulation of the walk-off effect in a lithium niobate crystal. (a) Reciprocal group velocity β_1 and group velocity dispersion β_2 as a function of the operation wavelength. (b) Dependence of the conversion efficiency on the interaction position within the nonlinear crystal. L_{crystal} is the length of the nonlinear crystal used in experiment. L_{eff} corresponds to the effective section of the crystal that is involved for the nonlinear conversion.

of the upconversion imager, allowing to identify incoming photons with different time delays [4]. In the femtosecond regime, the involved optical pulses are susceptible to the effects of temporal walk-off and group velocity dispersion (GVD) in the nonlinear crystal [6], which may affect the conversion efficiency and depth resolution of MIR upconversion 3D imaging.

In general, the walk-off effect leads to a temporal separation of the signal and pump pulses, which leads to the reduction of nonlinear conversion efficiency. The underlying behavior is manifested from the cross-correlation trace as varying the relative delay between the two interacting pulses. Specifically, an effective interaction length l_{eff} can be defined as the conversion efficiency drops to the half maximum, which is written as

$$l_{\mathrm{eff}} = \frac{\tau_{\mathrm{cross}}}{\left|\frac{1}{\nu_g^{(\mathrm{s})}} - \frac{1}{\nu_g^{(\mathrm{p})}}\right|} = \frac{\tau_{\mathrm{cross}}}{\left|\beta_1^{(\mathrm{s})} - \beta_1^{(\mathrm{p})}\right|} \ , \tag{2}$$

where τ_{cross} is the full-width at half maximum (FWHM) of the cross-correlation trace, $\nu_g^{(\mathrm{s,p})}$ correspond to the group velocities for the signal and pump pulses, and $\beta_1^{(\mathrm{s,p})}$ as the reciprocal of the group velocity denote the time delay per unit length. The peak width of intensity correlation τ_{cross} is measured to be 400 fs, which is related to the convolution between pulse durations of the signal and pump fields. The values of $\beta_1^{(\mathrm{s,p})}$ can be derived according to the Sellmeier equation for the refractive index of the nonlinear crystal. As shown in Supplementary Figure 2(a), $\beta_1^{(\mathrm{s})}$ at 3070 nm and $\beta_1^{(\mathrm{p})}$ at 1030 nm are calculated to be 7.315 ns·m^{-1} and 7.367 ns·m^{-1}, respectively. As a result, the value of l_{eff} is calculated to be 7.69 mm. In the experiment, the CPLN crystal covers the poling periods of 16-24 μm. The nonlinear crystal is designed to support a broadband spectral conversion over 3-5 μm [4]. For the specific operation wavelength at 3070 nm, the involved poling periods span from 19 to 21 μm, which permits a full acceptance angle of 30 degrees. By considering the 10-mm total length L_{crystal} of the crystal, the effective length L_{eff} for the nonlinear conversion is determined to be about 2.5 mm, which is much shorter than the effective interaction length l_{eff}.

We then analytically investigate the reduction of the conversion efficiency due to the walk-off effect. For the sake of simplicity, the intensity envelops of the signal and pump pulses are assumed to be Gaussian, which can be written as

$$\begin{aligned} I_{\mathrm{s}}(t) &= I_0^{(s)} e^{-4\ln 2 \times t^2/\tau_{\mathrm{s}}^2} , \\ I_{\mathrm{p}}(t - z d_{\mathrm{s,p}}) &= I_0^{(p)} e^{-4\ln 2 \times (t - z d_{\mathrm{s,p}})^2/\tau_{\mathrm{p}}^2} , \end{aligned} \tag{3}$$

where z represents the interaction position of the two-color pulses within the crystal, $I_0^{(\mathrm{s,p})}$ are the peak intensity of the signal and pump pulses, $\tau_{\mathrm{s,p}}$ are the FWHM pulse durations, and $d_{\mathrm{s,p}} = |\beta_1^{(\mathrm{s})} - \beta_1^{(\mathrm{p})}|$ is the walk-off parameter. At the position $z = 0$, both two pulses are temporally located at the center of the crystal, corresponding to the peak conversion efficiency. In the small-signal approximation, the conversion efficiency for the SFG process is proportional to the product of the signal and pump intensities. Therefore, the normalized conversion efficiency at the interaction position z can be described as

$$\eta(z) = \frac{\int_{-\infty}^{+\infty} I_{\mathrm{s}}(t) I_{\mathrm{p}}(t - z d_{\mathrm{s,p}}) dt}{\int_{-\infty}^{+\infty} I_{\mathrm{s}}(t) I_{\mathrm{p}}(t) dt} = e^{-4\ln 2 \frac{d_{\mathrm{s,p}}^2 z^2}{\tau_{\mathrm{s}}^2 + \tau_{\mathrm{p}}^2}} . \tag{4}$$

Given the parameters of $d_{\mathrm{s,p}} = 52$ ps·m^{-1}, $\tau_{\mathrm{s}} = 295$ fs and $\tau_{\mathrm{p}} = 270$ fs, the simulation result is shown in Supplementary Figure 2(b). The shaded area indicates the effective interaction length L_{eff} of 2.5 mm. The average conversion efficiency within L_{eff} is calculated to be 97%, which indicates a negligible walk-off effect in our experimental settings. In the case of using shorter pulses, the drop of the conversion efficiency will be more prominent. For instance, the reduction of the average efficiency reaches to 83 % with the presence of $\tau_{\mathrm{p}} = \tau_{\mathrm{s}} = 100$ fs.

In addition, the GVD plays an important role in the optical pulse broadening within a dispersive medium. As shown in Supplementary Figure 2(a), the dispersion parameter β_2 is calculated to be 0.247 ps^2·m^{-1} and -0.539 ps^2·m^{-1} at the wavelengths of 1.03 and 3.07 μm, respectively. Under the assumption of Fourier-transform-limited pulse pulses, the pulse duration at the output of the nonlinear crystal is given by the following formula

$$\tau_{\mathrm{out}} = \tau_{\mathrm{in}} \sqrt{1 + (4\ln 2 \frac{\beta_2 L_{\mathrm{crystal}}}{\tau_{\mathrm{in}}^2})^2} , \tag{5}$$

where τ_{in} and τ_{out} are the input and output pulse durations in FWHM, respectively. The output signal and pump pulses are calculated to be 299 fs and 271 fs, respectively. Therefore, the degradation of depth resolution due to the GVD effect is estimated to be less than 1%.

Supplementary Note 3: Spatial resolution of 3D imaging system

The MIR 3D imaging system is featured with a wide-field operation, where massive spatial elements at a precise gating time are recorded by a high-definition camera. The spatial resolution is determined by the setup parameters for the parametric upconversion imaging system. In the 4f imaging configuration shown in Supplementary Figure 1, the pump beam within the nonlinear

crystal acts as a soft aperture. At the presence of a Gaussian pump, only low-frequency components near the center of the Fourier plane can be efficiently upconverted, which renders the frequency conversion operation analogous to a low-pass spatial-frequency filter [3, 7]. In the experiment, the MIR beam is rapidly modulated by a spinning diffuser before illuminating the object, which favors to suppress the speckle pattens as usually observed in coherent imaging due to the high spatial coherence of the laser source [8]. Supplementary Figures 3(a) and (b) present the recorded images corresponding to the stationary and spinning states of the diffuser, respectively.

To quantitatively evaluate the incoherent imaging performance, the intensity distribution for the upconverted field can be expressed as [7]

$$I_{\mathrm{up}}(x', y', \Delta k_z) \propto \frac{8\pi^2 d_{\mathrm{eff}}^2 l^2 \lambda_{\mathrm{s}}^2}{n_{\mathrm{s}} n_{\mathrm{p}} n_{\mathrm{up}} c \varepsilon_0 f_2^2 \lambda_{\mathrm{up}}^4} P_{\mathrm{p}} \mathrm{sinc}^2 \left[\Delta k_z (-\frac{\lambda_{\mathrm{s}} f_1}{\lambda_{\mathrm{up}} f_2} x', -\frac{\lambda_{\mathrm{s}} f_1}{\lambda_{\mathrm{up}} f_2} y') \frac{l}{2} \right] \\ \times I_{\mathrm{object}}(-\frac{\lambda_{\mathrm{s}} f_1}{\lambda_{\mathrm{up}} f_2} x', -\frac{\lambda_{\mathrm{s}} f_1}{\lambda_{\mathrm{up}} f_2} y') \otimes \left[-\frac{2\pi w_{\mathrm{p}}^2}{(\lambda_{\mathrm{up}} f_2)^2} e^{-\frac{2(x'^2 + y'^2)\pi^2 w_{\mathrm{p}}^2}{(\lambda_{\mathrm{up}} f_2)^2}} \right] , \tag{6}$$

where $\lambda_{\mathrm{s,p,up}}$ denote the wavelengths for the signal, pump, and upconverted fields, $n_{\mathrm{s,p,up}}$ are the refractive indexes, d_{eff} is the effective nonlinear coefficient, c is the speed of light in vacuum, ε_0 is the vacuum permittivity, f_1 and f_2 are the focal lengths for the lens before and after the crystal, l is the interaction length, I_{object} is the intensity distribution at the object plane, P_{p} is the pump power, w_p is the beam size of the pump, and Δk_z is the phase mismatch in the propagation axis. In the phase-matching condition, the upconverted image is simply the convolution between the real-valued intensity of the rescaled object and the intensity distribution of the Fourier transform of the pump. The convolution relates to intensity rather than the amplitude results in an enhancing factor of $\sqrt{2}$ for the spatial resolution in comparison to that in the coherent imaging regime [8].

The spatial resolution is determined by the point spread function (PSF) of the imaging system. In the upconversion imaging architecture, the sharpness of the PSF depends inversely on the pump size as indicated in Eq. (6). Specifically, the spatial resolution $\mathcal{R}$ at the object plane can be given by the following definition [1]

$$\mathcal{R} = \frac{2 f_1 \lambda_{\mathrm{s}}}{\pi w_{\mathrm{p}}} \sqrt{2} \, . \tag{7}$$

For improving the spatial resolution, it is imperative to increase the transverse section of the nonlinear crystal and enlarge the beam size of the pump. In the experiment, the CPLN crystal is fabricated with a 2-mm thickness and 3-mm width. The crystal thickness is twice as that in previous demonstrations [4, 5], which favors to a two-fold improvement on the spatial resolution. Note that the fabrication of thicker PPLN crystals remains challenging for the current inverse poling technique. Indeed, the electric field needed to invert the electric-dipole orientation in the crystal is as large as about 22 kV·mm^{-1}, and is applied for only a few milliseconds. The voltage must be very carefully controlled so that the poled regions are properly produced with the desired structure. A thicker crystal leads a lager gap between the electrodes, which increase the difficulty to prepare a well-controlled ultra-high voltage source. Given the experimental settings of $f_1 = 50$ mm, $\lambda_s = 3.07$ μm, $w_p = 2$ mm, the spatial resolution is estimated to be about 69 μm.

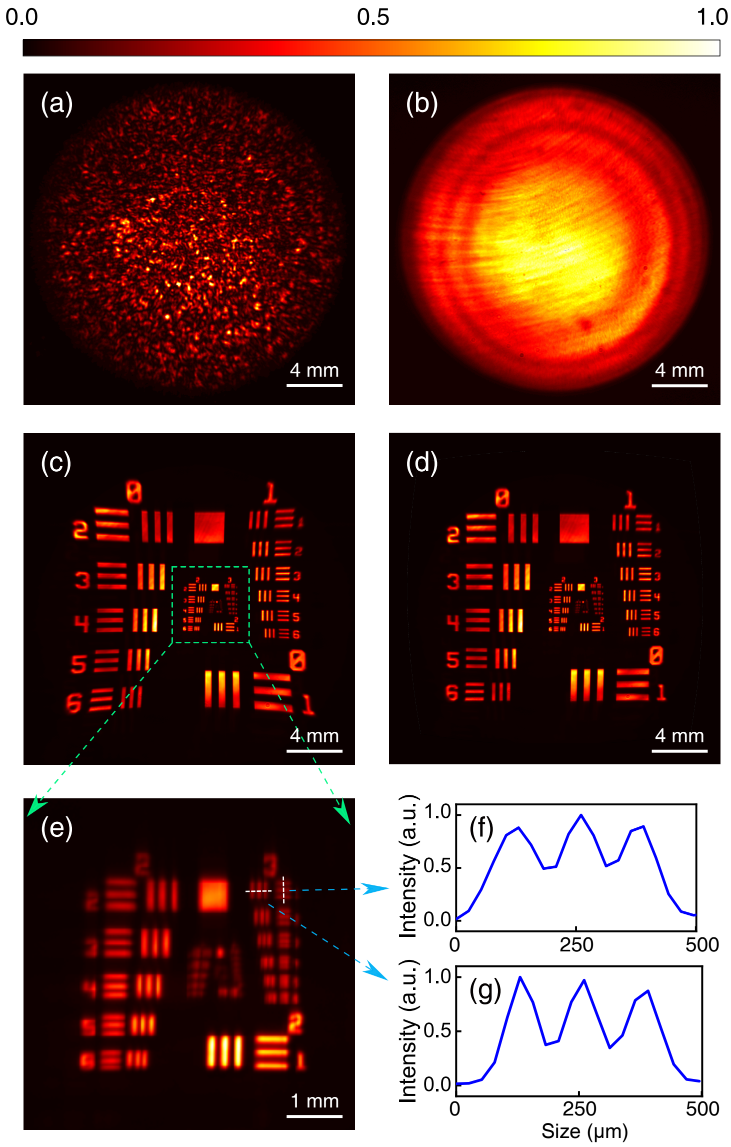

Supplementary Figure 3: Characterization of the spatial resolution for the wide-field imaging system. (a) Recorded speckle patten at the presence of a stationary diffuser. (b) Recorded image under a rapidly modulated illumination by a spinning diffuser. (c) Imaging performance for a USAF-1951 resolution target. (d) Corrected image for the pincushion distortion. (e) Zooming-in illustration for the central part in the test chart. Resolved bars for first element of group 3 in the test chart are denoted by the white dashed lines. The bar width is specified to be 62.5 μm. The cross sections along the horizontal and vertical directions are presented in (f) and (g), respectively.

In the experiment, a USAF-1951 resolution target is used to characterize the spatial resolution. The recorded image is shown in Supplementary Figure 3(c). The exhibiting pincushion distortion is ascribed to the radial-dependence magnification for an imperfect lens in 4f imaging system, instead of the intrinsic process for the nonlinear frequency conversion [4]. Supplementary Figure 3(d) presents the corrected image by using the lens correction filter available from common photo-editing softwares, such as Adobe Photoshop or GIMP. The central part in the test chart is zoomed

in for evaluating the resolution, as shown in Supplementary Figure 3(e). The cross sections for the first elements of group 3 are depicted in Supplementary Figures 3(f) and (g). The resolved bar width is 62.5 μm, closed to the predicated value according to Eq. (7). The achieved field of view is about one inch in diameter defined by the aperture of the optical lens, thus leading to 1.2×10^5 resolvable spatial elements. The number of resolvable elements in a single short is more than four-fold improvement over previous demonstrations for parametric upconversion imaging [1–5].

Supplementary Note 4: MIR volumetric imaging for stacked silicon wafers

The MIR light shows a low absorption for semiconductor materials, such as silicon and germanium. The time-resolved upconversion imager thus favors to retrieve the interior information, such as internal structures and surface reflectivity. Such a MIR volumetric imaging is particularly useful for non-invasive inspection of semiconductor chips. In the experiment, a proof-of-principle demonstration is performed with two stacked silicon wafters as a multiple-layer object. As shown in Supplementary Figure 4, the two wafers with thicknesses of d_1=630 μm and d_1=720 μm are separated by an air gap of d_{air}=1.02 mm. The MIR beam is illuminated from the top surface, and penetrates into various wafer/air interfaces. Supplementary Figure 4 presents the involved refections corresponding to the cross-correlation peaks shown in Figure 3 in the manuscript. Each reflection path is labeled with X_m^n, where X$\in$ {A, B} and m represent the surface group and surface number, and n indicates the nth reflection from the same surface according to an ascending order of the returning time.

The relative optical path between $A_1^{(1)}$ and $A_2^{(1)}$ is measured to be $2 \times n_{\mathrm{Si}} d_1 = 4.3$ mm, which enables us to deduce the refractive index of the silicon wafer $n_{\mathrm{Si}} = 3.41$. According to the Fresnel equations, the power reflectivity R and transmittance T at normal incidence for the silicon/air

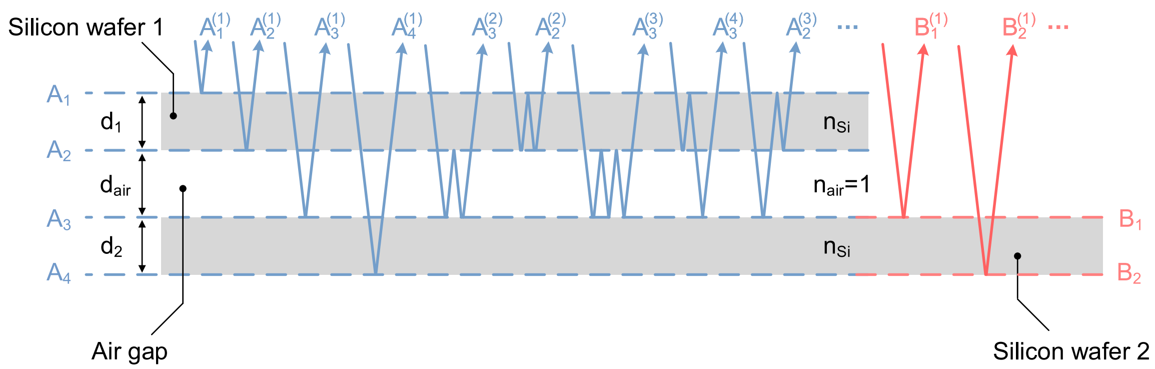

Supplementary Figure 4: Schematic diagram of the involved ballistic paths for the MIR probe photons within two stacked silicon wafers. The amplitude and delay for the reflected light can be measured by the time-resolved upconversion imager, which are manifested by a series of intensity-correlation peaks as presented in Figure 3 in the manuscript.

interface are given by

$$R = |\frac{n_{\mathrm{Si}}/n_{\mathrm{air}} - 1}{n_{\mathrm{Si}}/n_{\mathrm{air}} + 1}|^2 = 0.299,$$
$$T = 1 - R = 0.701\ . \tag{8}$$

Supplementary Table I presents the relative optical path and the normalized intensity for each reflective beam. A factor of 2 for the optical path is used based on the consideration of the double-pass configuration, which favors to infer the physical depth by simply correcting the refractive index of the propagating medium. In addition, the measured intensity for each intensity-correlation peak is also given, showing a good agreement with the theoretical one. It is worth noting that an identical optical path for $A_3^{(4)}$ and $A_2^{(3)}$ leads to a constructive interference, which is verified by the experimental observation.

Supplementary Table I: List of the involved reflections according to an ascending order for the optical paths.

Path tag	Relative optical path (×2)	Normalized intensity	Measured intensity
$A_1^{(1)}$	0	$I_0R = 1.000$	1.000
$A_2^{(1)}$	$n_{\mathrm{Si}}d_1$	$I_0T^2R = 0.490$	0.481
$A_3^{(1)}$	$n_{\mathrm{Si}}d_1 + d_{\mathrm{air}}$	$I_0T^4R = 0.242$	0.217
$A_4^{(1)}$	$n_{\mathrm{Si}}d_1 + d_{\mathrm{air}} + n_{Si}d_2$	$I_0T^6R = 0.119$	0.104
$A_3^{(2)}$	$n_{\mathrm{Si}}d_1 + 2d_{\mathrm{air}}$	$I_0T^4R^3 = 0.022$	0.021
$A_2^{(2)}$	$2n_{\mathrm{Si}}d_1$	$I_0T^2R^3 = 0.044$	0.041
$A_3^{(3)}$	$n_{\mathrm{Si}}d_1 + 3d_{\mathrm{air}}$	$I_0T^4R^5 = 0.002$	0.002
$A_3^{(4)}$	$2n_{\mathrm{Si}}d_1 + d_{\mathrm{air}}$	$I_0T^4R^3 = 0.022$	0.077*
$A_2^{(3)}$	$2n_{\mathrm{Si}}d_1 + d_{\mathrm{air}}$	$I_0T^4R^3 = 0.022$	
$B_1^{(1)}$	$d_1 + d_{\mathrm{air}}$	$I_0R = 1.000$	1.000
$B_2^{(1)}$	$d_1 + d_{\mathrm{air}} + n_{\mathrm{Si}}d_2$	$I_0T^2R = 0.491$	0.482

* Coherent superposition of two reflected fields from $A_3^{(4)}$ and $A_2^{(3)}$ with an identical optical path.

Supplementary Note 5: Algorithms for image denoising and reconstruction

In the low-light 3D imaging demonstration, the raw data is acquired by the EMCCD camera at various pump delays t_k. The pixel number at each frame is 1024×1024, and 750 frames are recorded to form the data cube $I(x, y, t_k)$. The step of depth scanning is set to be 20 μm, corresponding to a time delay of 133 fs. An algorithm is designed to retrieve the reflectivity and depth of the scene. Particularly, a pre-denoiser is needed to reveal the signal under severe noises in the photon-starved regime. To this end, the algorithm is operated with two main steps consisting correlation enhancement and edge detection. The numerical computation is performed by using MATLAB in a laptop with an Intel i5-8300H processor running at 2.30 GHz.

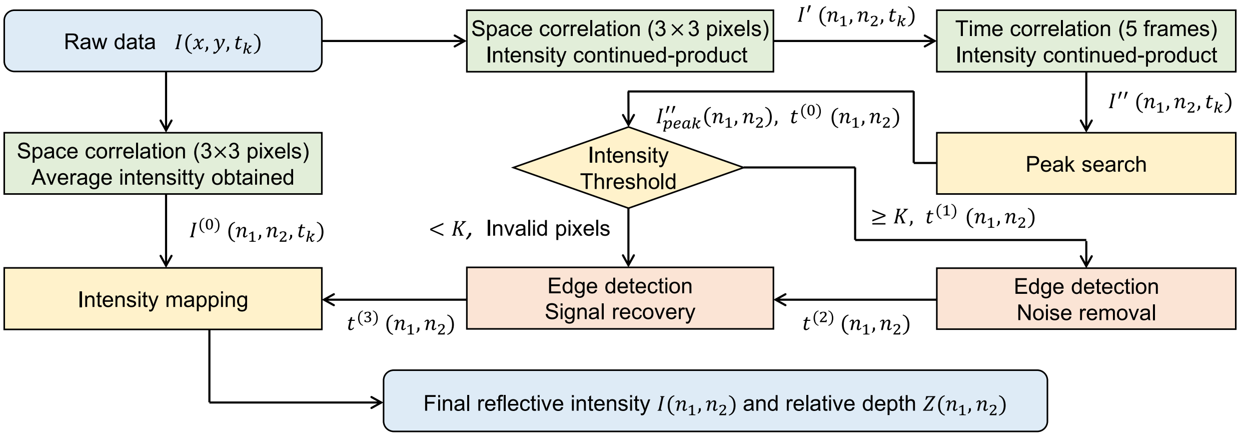

Supplementary Figure 5: Block diagram of the algorithmic process for the 3D imaging reconstruction. Each block is a subroutine and the arrow indicates the direction of the data flow.

Step 1. Correlation enhancement

- *Spatial correlation enhancement*

The foremost challenge in low-light-level imaging is to identify the signal overwhelmed by the severe noises [9, 10]. Indeed, the peak is hardly to be recognized from the measured intensity-correlation trace due to the low signal-to-noise ratio (SNR). The spatial correlation of a target is exploited to enhance the peak contrast. Specifically, each pixel value in the frame is replaced with the continued product of the 3×3 region consisting of the pixel and its eight neighboring elements. The operation is described with the following expression:

$$I'(n_1, n_2, t_k) = \prod_{x=n_1-1}^{x=n_1+1} \prod_{y=n_2-1}^{y=n_2+1} I(x, y, t_k) \ , \tag{9}$$

where $n_{1,2}$ are the spatial coordinates over the frame, and $n_{1,2} = 2, 3, \cdots, 1023$.

- *Temporal correlation enhancement*

The peak contrast of the intensity-correlation trace is subsequently enhanced in the axial dimension by performing another continued-product operation on the pixel intensities of every five sequential frames. The operation is explicitly given by

$$I''(n_1, n_2, t_k) = \prod_{t=t_k}^{t=t_{k+4}} I'(n_1, n_2, t) \ , \tag{10}$$

where t_k denotes the axial coordinate, and $k = 1, 2, \cdots, 746$.

- *Peak search*

The SNR of the correlation peak is significantly increased after the correlation-enhancement operation in the space and time domains, which facilitates to identify the peak position based on a simple maximum searching algorithm. The peak position is related to the depth information of

the interrogated surface. The involved peak-searching operation is expressed as

$$
\begin{aligned}
I''_{peak}(n_1, n_2) &= \max[I''(n_1, n_2, t_k)] \ , \\
t^{(0)}(n_1, n_2) &= \arg\max_{t_k}[I''(n_1, n_2, t_k)] \ ,
\end{aligned}
\tag{11}
$$

where $I''_{peak}(n_1, n_2)$ denotes the amplitude of the enhanced correlation-trace peak, and $t^{(0)}(n_1, n_2)$ is the corresponding axial position for the identified peak.

• *Intensity thresholding*

The correlation enhancement also favors to screen the noisy points arising from ambient scattering or dark counts, which helps to remove most of noisy pixels in the data cube. The background noise is typically associated with a relatively low intensity, hence an intensity threshold K can be applied for each pixel to label the invalid points. The operation is described as

$$
t^{(1)}(n_1, n_2) = \begin{cases} t^{(0)}(n_1, n_2), \text{ if } I''_{peak}(n_1, n_2) \geq K \ , \\ \text{Invalid value, otherwise} \ . \end{cases}
\tag{12}
$$

Step 2. Edge detection

• *Edge detection: Noise removal*

Furthermore, a edge-detection algorithm is implemented to remove the residual noisy pixels outside of the illuminated object. For a natural scene, the depth variation for the object surface is generally small, which is used to identify the boundary of the object. In the algorithm, the depth value at each spatial coordinate is compared to the neighboring pixels within the 7×7 sub-matrix. We count the number N of effective points, for which the depth difference is smaller than 30 depth steps. The depth information is valid only if $N \geq 25$.

Algorithm - Edge detection: Noise removal
1: Input: $t^{(1)}(n_1, n_2)$
2: for $n_1 = 4 : 1021$
3: for $n_2 = 4 : 1021$
4: if $t^{(1)}(n_1, n_2) \neq$ Invalid value
5: N = Countif (abs($t^{(1)}(n'_1, n'_2)$-$t^{(1)}(n_1, n_2)$)<30)
$n'_1 \in (n_1 - 3) : (n_1 + 3),\ n'_2 \in (n_2 - 3) : (n_2 + 3)$
6: end if
7: $t^{(2)}(n_1, n_2) = \begin{cases} t^{(1)}(n_1, n_2), \text{ if } N \geq 25 \\ \text{Invalid value, otherwise} \end{cases}$
8: end for
9: end for
10: Output: $t^{(2)}(n_1, n_2)$

- *Edge detection: Signal recovery*

In the photon-starved regime, the detected photons are very limited. There will inevitably be some points, for which the correlation-enhancement operation in the first step maybe insufficient to accurately identify the depth position of the intensity-correlation trace. The resulting abrupt estimates can be filtered by exploiting the feature of smooth edges in natural scenes. In the algorithm, the point labeled with "invalid value" is rechecked over the entire space. For each invalid pixel, we count the number M of valid elements within the neighboring 7×7 region. This point will be regarded to be informative only if $M \geq 25$. Meanwhile, the corresponding depth value is replaced by the average of the depths for those valid elements within the 7×7 region.

Algorithm - Edge detection: signal recovery
1: Input: $t^{(2)}(n_1, n_2)$
2: for $n_1 = 4 : 1021$
3: for $n_2 = 4 : 1021$
4: if $t^{(2)}(n_1, n_2) =$ Invalid value
5: $M =$ Countif $(t^{(2)}(n_1', n_2') \neq$ Invalid pixel)
$t = \sum t^{(2)}(n_1', n_2')$
$n_1^{'} \in (n_1 - 3) : (n_1 + 3), n_2^{'} \in (n_2 - 3) : (n_2 + 3)$
6: end if
7: $t^{(3)}(n_1, n_2) = \begin{cases} t/M, \text{ if } M \geq 25 \\ t^{(2)}(n_1, n_2), \text{ otherwise} \end{cases}$
8: end for
9: end for
10: Output: $t^{(3)}(n_1, n_2)$

- *Intensity mapping*

After the above operations, we have calculated the depth information $t^{(3)}(n_1, n_2)$ for each spatial point. The intensity distribution of the 3D scene is obtained from an intensity-mapping operation onto the data cube $I(x, y, t_k)$ as

$$I(n_1, n_2) = I(n_1, n_2, t^{(3)}(n_1, n_2)) \; . \tag{13}$$

Additionally, the depth map is given by

$$Z(n_1, n_2) = t^{(3)}(n_1, n_2) \; . \tag{14}$$

Note that the pixel interval of 26 μm and depth size of 20 μm should be taken into account as the spatial and axial scaling factors for properly illustrating the actual object.

Supplementary Video

Supplementary Video 1: All-around overview of the reconstructed stereoscopic object with an aim to illustrate the performance of the single-photon mid-infrared three-dimensional imaging. The mid-infrared illumination is set at the low-light level such that the average detected photon number per second is about one for each pixel.

[1] S. Junaid, S. C. Kumar, M. Mathez, M. Hermes, N. Stone, N. Shepherd, M. Ebrahim-Zadeh, P. Tidemand-Lichtenberg, and C. Pedersen, "Video-rate, mid-infrared hyperspectral upconversion imaging," *Optica* **6**, 702-708 (2019).

[2] M. Mrejen, Y. Erlich, A. Levanon, and H. Suchowski, "Multicolor time-resolved upconversion imaging by adiabatic sum frequency conversion," *Laser Photonics Rev.* **14**, 2000040 (2020).

[3] Y. Wang, J. Fang, T. Zheng, Y. Liang, Q. Hao, E. Wu, M. Yan, K. Huang, and H. Zeng, "Mid-infrared single-photon edge enhanced imaging based on nonlinear vortex filtering," *Laser Photonics Rev.* **15**, 2100189 (2021).

[4] K. Huang, J. Fang, M. Yan, E Wu, and H. Zeng, "Wide-field mid-infrared single-photon upconversion imaging," *Nat. Commun.* **13**, 1077 (2022).

[5] H. Zhang, S. Kumar, Y. M. Sua, S. Zhu, and Y.-P. Huang, "Near-infrared 3D imaging with upconversion detection," *Photonics Res.* **10**, 2760-2767 (2022).

[6] A. A. S, C. F. O'Donnell, S. C. Kumar, M. Ebrahim-Zadeh, P. Tidemand-Lichtenberg, and C. Pedersen, "Mid-infrared upconversion imaging using femtosecond pulses," *Photonics Res.* **7**, 783-791 (2019).

[7] A. Barh, P. J. Rodrigo, L. Meng, C. Pedersen, and P. Tidemand-Lichtenberg, "Parametric upconversion imaging and its applications," *Adv. Opt. Photonics* **11**, 952-1019 (2019).

[8] J. S. Dam, C. Pedersen, and P. Tidemand-Lichtenberg, "Theory for upconversion of incoherent images," *Opt. Express* **20**, 1475-1482 (2012).

[9] L. Kong, Q. Zhao, K. Zheng, H. Lu, S. Chen, X. Tao, H. Wang, H. Hao, C. Wan, X. Tu, L. Zhang, X. Jia, L. Kang, J. Chen, and P. Wu, "Noise-tolerant single-photon imaging with a superconducting nanowire camera," *Opt. Lett.* **45**, 6732-6735 (2020).

[10] D. Shin, F. Xu, D. Venkatraman, R. Lussana, F. Villa, F. Zappa, V. K. Goyal, F. N. C. Wong, and J. H. Shapiro, "Photon-efficient imaging with a single-photon camera," *Nat. Commun.* **7**, 12046 (2018).
